# Orbital controls on eastern African hydroclimate in the Pleistocene

**DOI:** 10.1038/s41598-022-06826-z

**Published:** 2022-02-24

**Authors:** Rachel L. Lupien, James M. Russell, Emma J. Pearson, Isla S. Castañeda, Asfawossen Asrat, Verena Foerster, Henry F. Lamb, Helen M. Roberts, Frank Schäbitz, Martin H. Trauth, Catherine C. Beck, Craig S. Feibel, Andrew S. Cohen

**Affiliations:** 1 Department of Earth, Environmental, and Planetary Sciences, Brown University, Providence, RI 02912 USA; 2 Biology and Paleo Environment, Lamont-Doherty Earth Observatory of Columbia University, Palisades, NY 10964 USA; 3 School of Geography, Politics & Sociology, Newcastle University, Newcastle Upon Tyne, NE1 7RU UK; 4 Department of Geosciences, University of Massachusetts Amherst, Amherst, MA 01003 USA; 5 Department of Mining and Geological Engineering, Botswana International University of Science and Technology, Private Bag 16, Palapye, Botswana; 6 School of Earth Science, Addis Ababa University, Addis Ababa, Ethiopia; 7 Institute for Geography Education, University of Cologne, 50931 Cologne, Germany; 8 Department of Geography and Earth Sciences, Aberystwyth University, Aberystwyth, SY23 3DB UK; 9 Botany Department, Trinity College Dublin, Dublin 2, Ireland; 10 Institute of Geosciences, University of Potsdam, 14476 Potsdam, Germany; 11 Geosciences Department, Hamilton College, Clinton, NY 13323 USA; 12 Department of Earth and Planetary Sciences, Rutgers University, Piscataway, NJ 08854 USA; 13 Department of Geosciences, University of Arizona, Tucson, AZ 85721 USA

**Keywords:** Palaeoclimate, Geochemistry

## Abstract

Understanding eastern African paleoclimate is critical for contextualizing early human evolution, adaptation, and dispersal, yet Pleistocene climate of this region and its governing mechanisms remain poorly understood due to the lack of long, orbitally-resolved, terrestrial paleoclimate records. Here we present leaf wax hydrogen isotope records of rainfall from paleolake sediment cores from key time windows that resolve long-term trends, variations, and high-latitude effects on tropical African precipitation. Eastern African rainfall was dominantly controlled by variations in low-latitude summer insolation during most of the early and middle Pleistocene, with little evidence that glacial–interglacial cycles impacted rainfall until the late Pleistocene. We observe the influence of high-latitude-driven climate processes emerging from the last interglacial (Marine Isotope Stage 5) to the present, an interval when glacial–interglacial cycles were strong and insolation forcing was weak. Our results demonstrate a variable response of eastern African rainfall to low-latitude insolation forcing and high-latitude-driven climate change, likely related to the relative strengths of these forcings through time and a threshold in monsoon sensitivity. We observe little difference in mean rainfall between the early, middle, and late Pleistocene, which suggests that orbitally-driven climate variations likely played a more significant role than gradual change in the relationship between early humans and their environment.

## Introduction

Understanding changes in eastern African hydroclimate during the Pleistocene is central to investigations of how humans evolved in a variable environment^1–8^. Over the Pleistocene, eastern African rainfall is thought to have undergone both secular and periodic changes driven by global cooling, evolving tropical sea surface temperature (SST) gradients, low-latitude insolation forcing, and glacial–interglacial cycles^3,9–17^. Each of these forcings has specific implications for the nature and timing of eastern African rainfall changes, which in turn yield predictions for the environmental changes experienced by our hominin ancestors. However, a lack of long datasets capable of resolving orbital cycles (10^3^–10^5^ years) limits our understanding of the relative influences of global climate forcings on the Pleistocene evolution of tropical eastern African rainfall, as well as the effects of paleoenvironmental change on early humans.

Varying seasonal insolation, controlled by the Earth’s orbital precession and eccentricity, causes changes in the differential heating of the African continent and oceans, driving fluctuations in the East African Monsoon strength^18,19^. 21-kyr cycles in monsoonal rainfall that result from this process are well-documented in eastern African climate records^9,11,20–26^, and their varying amplitude has been argued to have played a pivotal role in human evolution^6,7,27^. Coupled changes in the Earth’s carbon cycle and atmospheric greenhouse gas concentrations, global temperatures, and high-latitude glacial–interglacial cycles are also thought to play a critical role in eastern African climate evolution^3,4,28^, and long-term variations in these processes may have contributed to the development of bipedalism and other traits^29^. For instance, soil carbonate isotope (δ^18^O_sc_) records indicate gradual drying in northern and tropical Africa^30,31^, attributed to global cooling and ice-volume growth through the Pleistocene. Records of dust from the eastern Atlantic and the Mediterranean and Arabian Seas suggest transitions from 21- to 41- to 100-kyr periodicity over the Plio-Pleistocene, with shifts toward drier conditions and increased variability starting between 3500 and 2500 ka (onset and gradual intensification of Northern Hemisphere glaciation) and at 1000 ka^3,4^ (mid-Pleistocene Transition, MPT), matching transitions in the marine oxygen isotopic record of global ice volume^32^. However, recent accumulation rate corrections^33^ and time series analyses^12^ suggest different timings of aridification and a stronger influence of low-latitude insolation. Furthermore, strengthening of zonal SST gradients in the tropical Pacific beginning at ~1700 ka^34^ is thought to have weakened convection over eastern Africa, contributing to regional drying^16^. To date, despite the paleoanthropological significance of eastern Africa, the relative importance of low- and high-latitude climate forcings on the region’s rainfall history remain poorly constrained.

The Hominin Sites and Paleolakes Drilling Project (HSPDP) recovered sediment drill-cores that record the environmental history of key hominin fossil locales in Ethiopia and Kenya^35–37^. The cores allow us to develop and compare multiple long, high-resolution records of regional hydroclimate within a set of key time windows to elucidate the forcings and mechanisms of climate change in the region. Here we present a new record of the hydrogen isotopic composition of precipitation (δD_precip_) from compound-specific analyses of terrestrial leaf waxes—a novel and powerful proxy for processes related to rainfall^38^—preserved in middle to late Pleistocene sediments from the Chew Bahir Basin, Ethiopia. This is compared with an existing record of the early Pleistocene from the adjacent Omo-Turkana Basin^24^ to evaluate changing trends and rhythms in regional hydroclimate, as well as the relative influences of high- and low-latitude forcings during intervals of the early and middle to late Pleistocene.

**Figure 1:**
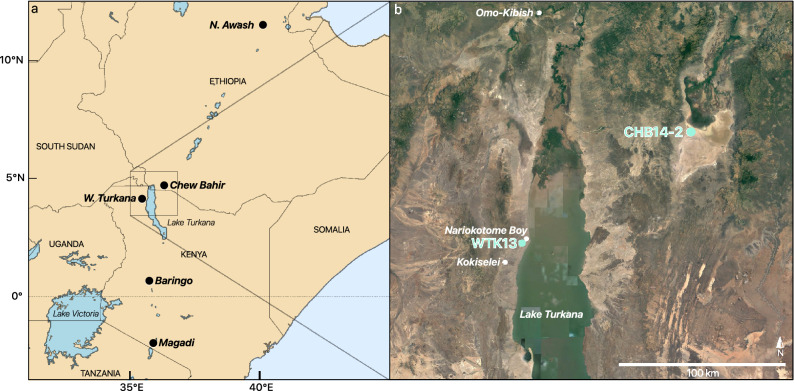
(**a**) East African Rift System study area map, including HSPDP sites and major rift lakes, generated in Python 3.8; (**b**) Ethiopian and Kenyan locations of the two paleolake sediment drill cores, WTK13 and CHB14-2, included in this study with Omo-Kibish and Nariokotome Boy hominin sites and the Kokiselei site of the first evidence for Acheulean hand axes^48^. Map generated in Google Earth Pro 7.3.3.

**Figure 2:**
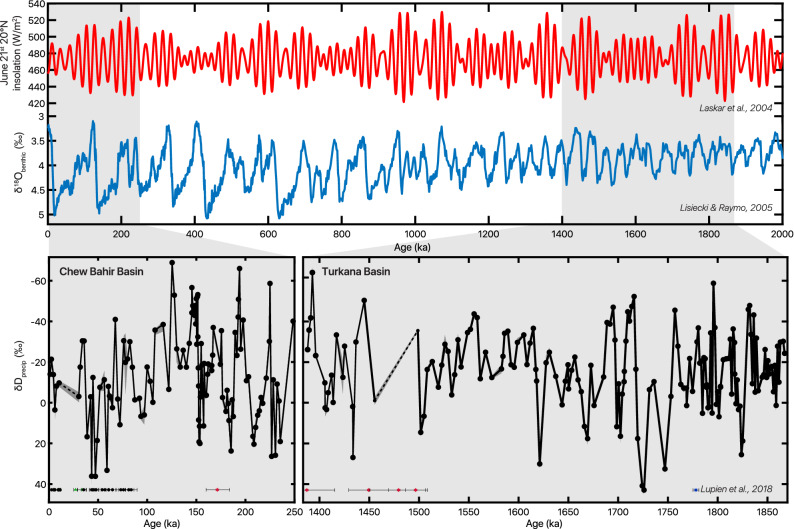
δD_precip_ records corrected for vegetation, ice volume, and geographic effects (Fig. S4) from CHB14-2 and WTK13 in the context of two million years of zonal mean 20° N June 21st insolation^65^ (red) and the benthic foraminifera δ^18^O stack^32^ (blue). Sampling gaps greater than half of a precession cycle (~10 kyr) are represented with dashed lines and analytical error on δD_wax_ measurements in shading. Age constraints for CHB14-2 and WTK13^24,45^ with 1σ analytical error depicted along bottom with symbol indicating dating technique (green triangle = ^14^C; black circle = OSL; red star = ^40^Ar/^39^Ar; blue square = magnetostratigraphy).

The HSPDP core locations lie in the East African Rift System (Fig. 1a), host to many famous hominin fossil sites^39–41^. We generated a new hydroclimate record derived from the hydrogen isotopic composition of terrestrial leaf waxes (δD_wax_) preserved in paleolake deposits from Chew Bahir, southern Ethiopia (duplicate drill cores HSPDP-CHB14-2A and -2B merged to composite core^42,43^, hereafter CHB14-2). Coring site CHB14-2 (4° 45′ 40″ N, 36° 46′ 00″ E) is located in the Chew Bahir Basin, just northeast of the Omo-Turkana Basin (Fig. 1b). Today, the southern part of the basin floor is mostly occupied by a saline mudflat. The composite core extends from ~620 ka to present with age constraints based on ^40^Ar/^39^Ar dating of tephra, optically stimulated luminescence (OSL), radiocarbon dating, and tephrostratigraphic correlations^44^. We analyzed waxes spanning the interval from ~250 ka to present-day and synthesized this new dataset with a published record from West Turkana, Kenya^24,45^ (1900–1400 ka; HSPDP-WTK13-1A, hereafter WTK13) located ~100 km from CHB14-2 (4° 6′ 35″ N, 35° 52′ 18″ E). The age model for WTK13 is based on tephrochronology and magnetostratigraphy and includes very conservative tuning of δD_precip_ with no impact on the dominance of orbital precession in the spectral properties^24^ (Fig. S1). Our combined datasets provide a regional hydroclimate record that represents a total span of ~750 kyr during the period 1900 ka to present, with an average sampling resolution of ~3 kyr within each record (Fig. 2).

The combined WTK13 and CHB14-2 data record key intervals when our genus, *Homo*, was evolving, developing new technologies, and dispersing within and out of Africa^46^. The Omo-Turkana Basin contains over 100 archaeological sites and 500 fossil finds^47^, including the earliest and most complete skeletons of *H. rudolfensis* and *H. erectus*. The ~1900–1400 ka interval spanned by WTK13 witnessed the development of Acheulean stone tools (earliest evidence for advanced hand axes at ~1760 ka at Kokiselei^48^, Fig. 1b), the evolution of *H. erectus* (including the Nariokotome Boy skeleton at ~1600 ka^49^, Fig. 1b), and what is thought to be the earliest hominin dispersal out of Africa^50^. The first eastern African evidence of our species, *H. sapiens*, is dated to ~233 ka at Omo Kibish in the Omo-Turkana Basin^51^, 100 km northwest of Chew Bahir (Fig. 1b). The past ~250 kyr, recorded in CHB14-2, not only encapsulates human morphological changes, but also social, technological, linguistic, and cultural development, and the dispersal of modern *H. sapiens* out of Africa^42,43,52^. These new traits spread to the rest of the world during this interval, and thus, this Turkana-Chew Bahir region may have served as a critical landscape for the development of our ancestors over the Pleistocene. This study, situated within the broader context of the aims of HSPDP (Fig. 1a), provides crucial insight into the nature of environmental change and the potential effects on hominins and other large mammals on the landscape.

Many paleoenvironmental indicators are very sensitive to basin-scale geological processes, limiting the ability for inter-basin comparison. However, δD_wax_ is primarily controlled by δD_precip_^53^, which, in tropical Africa, is dominantly driven by regional atmospheric dynamics that govern rainfall amount^54,55^. A variety of observational^55,56^, modeling^57^, and paleoclimate^14,24,58–60^ studies have revealed δD_precip_ to be very sensitive to changes in eastern African paleohydrology on orbital timescales. Although we recognize that δD_precip_ can be influenced by a variety of other processes such as moisture source and transport, and a variety of convective processes including the location of convective cells^61^, we interpret δD_precip_ as a qualitative indicator of rainfall amount, consistent with previous studies in the region^14,24,54,59,61,62^. We directly compare δD_precip_ between different sedimentary archive sites and time intervals to understand large-scale climate processes.

C_3_ and C_4_ metabolic processes influence the apparent fractionation between δD_wax_ and δD_precip_, but carbon isotopic compositions of the same leaf wax compounds (δ^13^C_wax_; Fig. S2) help estimate vegetation type and correct δD_wax_ to δD_precip_ (Fig. S3 and S4). While uncertainties exist in the biosynthetic fractionation factor, this correction has minimal influence on the trends and patterns in the precipitation record because the isotopic range in δD_precip_ is vastly larger than the potential C_3_–C_4_ effect. We also correct for geographic differences in δD_precip_ between WTK13 and CHB14-2 using δD_wax_ and δ^13^C_wax_ measurements from late Holocene sediment within each basin to estimate regional δD_precip_ (Fig. S4). We conduct a series of time series analyses to detect changes in the trends and rhythms of δD_precip_ and eastern African climate variability.

## Results

### Leaf wax biomarker record

The hydrogen isotopic composition of long-chain leaf waxes (*n*-C_26_, *n*-C_28_, and *n*-C_30_ alkanoic acids) are strongly correlated in CHB14-2 (C_28_–C_26_: r^2^ = 0.72, n = 100, *p* << 0.01; C_28_–C_30_: r^2^ = 0.9, n = 117, *p* << 0.01) demonstrating these compounds were derived from a common source and record similar climate processes. Despite previous work that found that *n*-C_28_ may be produced in the lake water column in some lakes^63^, the strong correlation between long-chain compounds indicates that *n*-C_28_ is representative of terrestrial land plants in this basin. As *n*-C_28_ is the most abundant long chain *n-*acid, determined by Average Chain Length (ACL) calculation (28.4), resulting in lower analytical error, we use the hydrogen isotopic ratio of C_28_ *n-*acid for all analyses of climate variability for both sites. The Carbon Preference Index (CPI) is a measurement of degradation of the organic compounds in the sediment, where a high even:odd chain length signifies good preservation of alkanoic acids, and a ratio of 1 signifies full degradation^64^. The CPI in CHB14-2 is acceptable (mean: 2.8; minimum: 1.5), and to further demonstrate the lack of degradation effect on isotope analyses, we compare CPI and δD_wax_ to find an insignificant correlation (r^2^ = 0.002, n = 125, *p* > 0.05). In CHB14-2, δD_wax_ ranges from − 164.6 to − 68.7‰.

δ^13^C_wax_ averages − 23.8‰ in CHB14-2, and ranges from − 19.9 to − 30.8‰ with one outlier at − 16.8‰ (Fig. S2). The corrected δD_precip_ record, based on the δ^13^C_wax_ data, ranges from − 68.9 to 36.2‰ and closely tracks δD_wax_ (Fig. S3 and S4).

### Trend, variability, and spectral properties

Neither of the δD_precip_ records show significant linear trends towards wetter or drier conditions within the time intervals they span individually or together, nor is there a large difference between the WTK13 and CHB14-2 study intervals (< 2‰ offset in δD_precip;_ Fig. 2).

**Figure 3:**
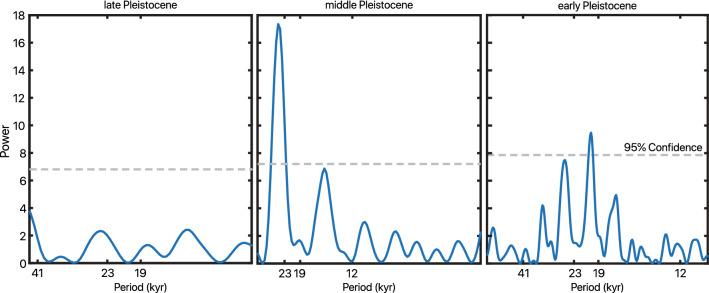
Lomb-Scargle spectral analyses for unevenly sampled data of δD_precip_ from the early (1900–1500 ka), middle (250–130 ka), and late (130–0 ka) Pleistocene. Precession-band 19- and 23-kyr periodicities lie above the 95% confidence line (dashed grey) in the early and middle Pleistocene. Frequency distribution is plotted from ½× the Nyquist frequency as the high-frequency cutoff to 1/3 of the total length of interval as the low-frequency cutoff, thus the differing x-axes of the three windows depend on the resolution and length of the specific interval.

**Figure 4:**
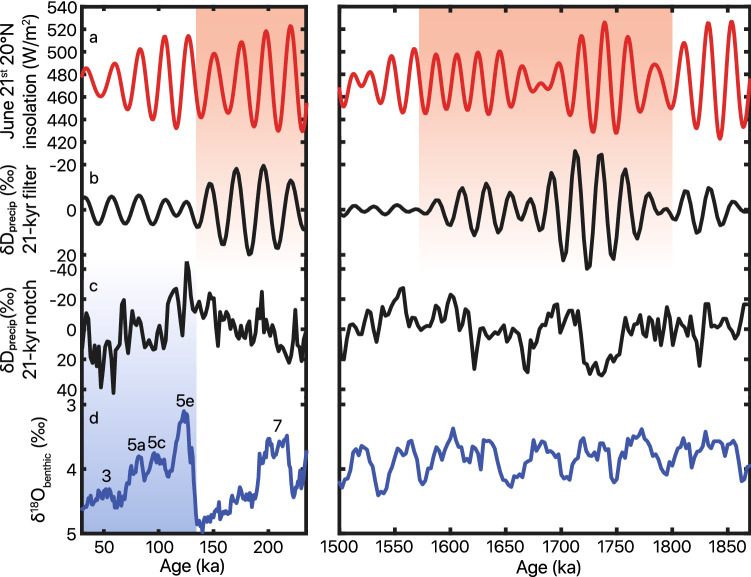
Gaussian 21-kyr ± 5-kyr band-pass (**b**) and notch (**c**) filtering of the δD_precip_ study intervals truncated to 1870–1500 ka and 250–30 ka to omit low sampling resolution sections. June 21st zonal mean 20° N insolation^65^ (**a**) plotted and highlighted in light red demonstrate similarity with high- and low-amplitude variability packets in gaussian band-pass filtered δD_precip_. Our selection of June 21st insolation at 20°N is based on observations from latest Pleistocene and Holocene records demonstrating the sensitivity of eastern African precipitation to this date and latitude^14,61,84,88^. We note that the chronologies for the CHB14-2 and WTK13 records are too imprecise to determine the phase of the response of δD_precip_ to orbital forcing; however, the choice of latitude and season does not influence our spectral analyses nor other results. Benthic foraminifera δ^18^O stack^32^ (**d**) plotted with recent interglacial MIS’s and highlighted in light blue to demonstrate similarily with late Pleistocene notch-filtered (precession-band periodicities removed) δD_precip_. Means were removed in both band-pass- and notch-filtered data to feature changes in variability.

Our δD_precip_ records contain high-amplitude oscillations of up to ~100‰. Lomb-Scargle periodogram analysis demonstrates spectral density at ~21 kyr in the early and middle Pleistocene intervals (1900–1500 ka and 250–130 ka) but no significant spectral properties in the late Pleistocene within the bounds of robust frequency detection (Fig. 3). Gaussian 21-kyr band-pass filtering of δD_precip_ in the two study intervals supports the spectral analysis findings of strong precession influence in the early and middle Pleistocene, and reveals that this precession-band variation is greatly diminished in the late Pleistocene (Fig. 4). After applying a notch filter to remove variability associated with the ~21 kyr band, we observe gradual D-enrichment from Marine Isotope Stage (MIS) 5 (~125 ka) until the beginning of MIS 2 (~30 ka). This trend coincides with increasing benthic foraminiferal δ^18^O, suggesting that shifts in the late Pleistocene δD_precip_ covary with glacial–interglacial cycles (Fig. 4c,d).

## Discussion

Our δD_precip_ records indicate eastern African rainfall experienced high-amplitude, orbitally-driven wet/dry cycles during long intervals of the early, middle, and late Pleistocene. Variability in the early Pleistocene 1900–1400 ka and middle Pleistocene (230–150 ka) intervals is dominated by orbital precession, with strong 21-kyr cycles in δD_precip_ (Figs. 3, 4), as well as 100-kyr eccentricity-band amplitude modulation (Fig. S5). Ice volume and associated global climate processes varied primarily at the 41-kyr period during the early Pleistocene and had a saw-tooth pattern and 100-kyr periodicity in the middle Pleistocene^32^, yet we see no robust signal of obliquity in the early Pleistocene (Fig. 3) nor visual similarity between δD_precip_ and ice volume through most of the record (Fig. 2). Instead, eastern African rainfall varied primarily at a 21-kyr precession rhythm (Fig. 3) with modulation of that variability by eccentricity into high- and low-amplitude packets (Fig. S5), in sync with low-latitude summer insolation forcing^65^ during the early to middle Pleistocene.

We observe no difference in mean values of δD_precip_ between the WTK13 and CHB14-2 records, suggesting remarkable long-term stability in eastern African rainfall during the Pleistocene. The similar lack of trend in the eastern Africa soil carbonate δ^18^O compilation^31^ suggests that the Omo-Turkana and Chew Bahir Basins, despite their aridity relative to surrounding basins, capture regional paleoclimate changes, especially because of the large-scale integrative nature of the leaf wax biomarker proxy. The long-term hydroclimate stability occurs despite evidence for regional C_4_ grassland expansion^66–72^, supporting recent work suggesting that declining atmospheric CO_2_, rather than hydroclimate, plays a dominant role in C_4_ grass expansion in Africa^73,74^.

Orbital-scale vegetation change, though, covaries with hydroclimate variations in intervals throughout the Quaternary^24,59,75^, and we observe substantial changes in the amplitude of orbital-scale variability within each of our records. Band-pass filtering of the precession signal in our δD_precip_ records isolates packets of high-amplitude variability that generally align with high orbital eccentricity and intervals with the strongest seasonal insolation forcing (Fig. 4 and S5). Although not every high eccentricity interval produces high-amplitude δD_precip_ oscillation (i.e., 1900–1800 ka), this result further suggests a dominant role for precession-driven seasonal insolation change in controlling eastern African rainfall during the early and middle Pleistocene.

Our findings are supported by records that indicate a dominant role for orbital precession in controlling African climate history, particularly in subtropical and northern Africa^9,20,22–26,76^. For instance, sapropel records from the Mediterranean indicate precessional insolation forcing has been a dominant driver of northeast African rainfall throughout the Plio-Pleistocene^9^. Our results are also consistent with some paleoclimate model simulations^17^, though others predict a stronger role for atmospheric greenhouse gases in eastern equatorial Africa^76^ than suggested by our records. Synchronized pulses of deep lakes in multiple East African Rift basins have been suggested to occur during intervals of high eccentricity^5^. Our δD_precip_ records indicate that high eccentricity intervals were times of much wetter, as well as much drier, conditions (Fig. S5), and the alternation between extreme endmembers suggested by our data could drive selection for generalist or adaptable traits in early humans^27,77,78^.

Despite the dominant role of orbital precession in our records, our δD_precip_ suggests global climate conditions became increasingly influential on tropical African rainfall between the middle and late Pleistocene after the last interglacial at ~130 ka. After removing precessional periodicity from our data, we observe a trend toward drier conditions from MIS 5e (when ice volume levels were similar to the Pliocene^32^) until the Last Glacial Maximum (LGM; Fig. 4). Previous work has documented strong influences of ice volume on eastern African climate during the latest Pleistocene, such as drying over most of the region during the LGM^43,79^. A ~210 kyr-long δD_wax_ record from the Gulf of Aden also documents strong precession-band rainfall variations during MIS 5, 6, and 7 superimposed on alternating humid and arid conditions that track ice volume^14^. This mixture of signals of insolation and ice volume in the Gulf of Aden potentially results from its more northern location or the larger area of leaf wax supply to this marine record. However, a dust record from the Mediterranean, which is thought to record Northeast African monsoon strength, also demonstrates precession-band fluctuations throughout the last 3000 kyr until a large, 100-kyr, sawtooth-shaped excursion begins in MIS 5e^12,76^.

Climate model simulations suggest strong atmospheric teleconnections between eastern African rainfall and the northern high latitudes^80^. One potential mechanism for the influence of late Pleistocene glacial–interglacial cycling in tropical Africa could be that cooling in the northern high latitudes is advected by the westerlies into Eurasia, which enhances the boreal winter Arabian anticyclone^80^. Northerly winds originating from this circulation advect cool and dry air over eastern Africa, suppressing boreal fall and winter rainfall. These simulations rely on freshwater hosing to cool the northern high latitudes and are therefore not directly analogous to the Northern Hemisphere glaciation cycles. However, these simulations demonstrate an atmospheric mechanism linking eastern African rainfall and northern high latitude climate via Eurasia that could apply on longer timescales.

Our δD_precip_ data suggest that low-latitude insolation forcing controls much of the long-term variability in eastern African rainfall, including during the middle Pleistocene when ice volume changes were large. However, ice volume fluctuations leave distinct signals from 130 ka to the present (Fig. 4) and there is also a stark lack of similarity between δD_precip_ and precession (Fig. 3) and eccentricity modulation (Fig. S5) during this time. We suggest that this arises due in part to the relative strengths of high- and low-latitude forcings. High-amplitude seasonal insolation forcing under high orbital eccentricity causes strong, periodic changes in eastern African rainfall^17^. However, when ice volume fluctuations strengthen and insolation forcing weakens, such as occurred from ~130 ka to the present, ice volume changes can emerge as a strong influence on eastern African hydroclimate. The shift from insolation-driven to ice volume-driven fluctuation at ~130 ka in our record suggests a nonlinear sensitivity of eastern African rainfall to seasonal insolation forcing and to high-latitude-driven climate change at this orbital time scale. This varying sensitivity to forcings of variable amplitude may reconcile the large number of records that document eastern African aridity during the LGM^43,58,79,81–86^, when ice volume changes were large and eccentricity was particularly low, against the longer Pleistocene records that show a dominant control of orbital precession on eastern African rainfall. This hypothesis may further explain the absence of 41-kyr cycles in African rainfall during the early Pleistocene, as ice volume changes were generally small compared to those during the late Pleistocene. Climate modeling experiments have suggested threshold responses of tropical climate to Northern Hemisphere ice volume changes, due to shifts in the position of westerly jets and their ability to perturb the tropical atmospheric circulation^87^. Additionally, threshold-like responses of African hydroclimate to insolation have been documented^61,87^ and attributed to various processes, including feedbacks involving vegetation, soil moisture, and SST^43,58,88^. The interaction of these nonlinear responses to high- and low-latitude climate drivers may have triggered shifts in sensitivity, depending on the relative strengths of each forcing.

Both orbital-scale variability and secular trends in eastern African climate have been postulated as drivers of hominin evolution and dispersal^3,7,42,43,77,89^. Our proxy records indicate that orbital-scale variability (up to 100‰ in a single precession cycle) is much larger than the long-term mean change occurring since ~2000 ka. Extremely high-amplitude fluctuations occurred in the region during critical times of early hominin evolution in eastern Africa and potentially promoted an environment that favored behavioral and morphological plasticity or adaptability in our ancestors^8,27^.

## Methods

### Geochemical analyses

We analyzed the isotopic composition of terrestrial leaf wax biomarkers preserved in sediment from composite core HSPDP-CHB14-2 (hereafter termed CHB14-2^42^) archived at the National Lacustrine Core Repository. Plants produce epicuticular waxes to shield leaf surfaces from evaporation and physical damage^90^. These waxes may be ablated and transported by eolian and fluvial processes to lakes, where they are preserved in sediment over geological time. The waxes include long-chain *n*-alkanoic acids, which we use to reconstruct water isotope compositions. Lipid extraction, purification, and isotopic analytical procedures^91^ were performed at Brown University. Lipids were extracted from freeze-dried and homogenized sediment using a DIONEX Accelerated Solvent Extractor 350 with dichloromethane:methanol (9:1). The total lipid extract was separated into neutral and acid fractions via aminopropylsilyl gel column with dichloromethane:isopropanol (2:1) and ether:acetic acid (24:1). The acid fraction was then methylated using acidified methanol, and the resulting fatty acid methyl esters (FAMEs) were purified using a silica gel column. Relative concentrations of the FAME chain lengths were quantified using an Agilent 6890 gas chromatograph (GC) equipped with a HP1-MS column (30 m × 0.25 mm × 0.25 µm) and flame ionization detector (FID).

Hydrogen isotopes (δD_wax_) were measured using an Agilent 6890 GC, equipped with HP1-MS column (30 m × 0.32 mm × 0.25 µm), coupled to a Thermo Delta Plus XL isotope ratio mass spectrometer (IRMS) with a reactor temperature of 1445 °C, although some of the samples from the CHB14-2 core were analyzed with a Thermo Delta V Plus IRMS using the same conditions. On both instruments, D/H ratios were measured in triplicate using H_2_ as an internal standard with He as the carrier gas, and corrected using a known FAMEs lab standard. Carbon isotopes (δ^13^C_wax_) from CHB14-2 and the late Holocene analogues were measured at Brown University with these same procedures on the Thermo Delta V Plus GC-IRMS with a reactor temperature of 1100 °C. Isotope ratios were corrected for the added methyl group (δD_MeOH_ =  − 123.7‰ and δ^13^C_MeOH_ =  − 36.62‰). We report δD_wax_ relative to Vienna Standard Mean Ocean Water (VSMOW) and δ^13^C_wax_ relative to Pee Dee Belemnite (PDB) in per mil (‰) notation.

We successfully analyzed 125 samples (out of 143 samples) for δD_wax_ and 92 samples for δ^13^C_wax_ from the CHB14-2 composite core. The sediment samples integrate up to 4 cm (~80 years^44^) and have a mean temporal resolution of ~1.75 kyr since 250 ka. Hydrogen isotopic analyses of the FAMEs standard had a standard deviation (1σ) of 3.2‰ and the H_3_ factor was 1.76 ppm/nA. For hydrogen, 56 samples were run in triplicate (average 1σ = 1.5), 20 in duplicate (average difference = 2.3‰), and 49 as single injections due to limited concentration. For carbon, all samples were measured in duplicate, with an average FAMEs standard 1σ of 0.25 and average intra-sample difference of 0.14‰. Five samples were removed from further analysis because they lie between two ages that constrain a potential sedimentary hiatus or dramatic reduction in sediment accumulation rate around the LGM from ~30–10.5 ka^42–44^.

### Isotopic corrections

A series of corrections to δD_wax_ were performed to convert values to δD_precip_ (Fig. S4). Once all corrections were made, one outlier (outside 3 standard deviation units) was removed from the WTK13 record.

#### Vegetation correction

C_3_ trees and C_4_ grasses fractionate hydrogen to different degrees during leaf wax synthesis due to differing metabolic pathways and plant physiologies. This causes different apparent fractionations between leaf waxes and precipitation (ε_wax-P_), which can affect paleoclimate records based on δD_wax_ if vegetation changes^38^. We calculated a ‘vegetation correction’ based upon δ^13^C_wax_ values (Fig. S2) to correct δD_wax_ for these differences^91^. We use δ^13^C_wax_ endmember values for C_3_ and C_4_ plant types previously described from a Omo-Turkana Basin outcrop^92^, in which the δ^13^C of *n*-C_30_ acids is − 32.9‰ for the C_3_ endmember and the δ^13^C of *n*-C_30_ acid is − 19.0‰ for the C_4_ end member. We adjust these values to account for observed differences between *n*-C_30_ and *n*-C_28_ acids^93^, thereby using − 32.15‰ and − 20.63‰ as the C_3_ and C_4_ endmembers. Samples with δ^13^C_wax_ values more enriched than this C_4_ endmember value were treated as 100% C_4_. After applying this C_3_/C_4_ mixing model to our δ^13^C_wax_ data, we then applied ε_wax-P_ values of − 112.8‰ and − 124.5‰ for C_3_ and C_4_ vegetation with a 25‰ correction for C_27_ *n*-alkane to C_28_ *n*-acid^38,91,94^ to correct for ‘vegetation effects’ on δD_wax_ and estimate δD_precip_ (Fig. S3).

Because not all δD_wax_ measurements have a corresponding δ^13^C_wax_ measurement, typically due to concentration limitations, we used AnalySeries^95^ to mathematically resample the δ^13^C_wax_ data to δD_wax_ resolution to obtain a δD_precip_ record with the same resolution as δD_wax_. In Fig. S2 we demonstrate that this does not have a meaningful impact on our results as the corrections are much smaller than the hydroclimate signals in δD_wax_ and δD_precip._ We show the CHB14-2 δD_precip_ record with and without the additional resampled δ^13^C_wax_ corrections to demonstrate that the difference between the δD_wax_ and the empirically derived δD_precip_ is negligible.

#### Ice volume correction

We use the benthic δ^18^O stack^32^ to estimate past ocean water isotopes to correct the δD_precip_ for different source water δD^91^. Age uncertainty in our records and in the LR04 stack limits our ability to precisely align the two, so we average the stack δ^18^O in each study interval, anomalize that value to late Holocene, and convert it to δD based on the meteoric water line. We then apply this anomaly to each study interval to obtain an ice volume-corrected signal of δD_precip_ (Fig. S4).

#### Geographic correction

δD_wax_ and δ^13^C_wax_ measurements of late Holocene analogue sediment (Table S1) lets us obtain δD_precip_ measurements from both sites. One sample from the Chew Bahir Basin^96^ and 12 averaged samples from the Omo-Turkana Basin^85^ were used to represent the late Holocene (last 5 kyr) leaf wax isotope signature of each region (Table S1). Our late Holocene analogue measurements of δD_precip_ are similar to modeled precipitation isotope data^97^, indicating that we have appropriately captured the differences between study sites. We anomalized the Chew Bahir measurements to Turkana δD_precip_. This “geographic” correction (12‰) was then added to the mean of the CHB14-2 record (Fig. S4) to produce the fully corrected eastern African δD_precip_ Pleistocene record (Fig. 2).

### Time series analyses

We analyzed the linear trends within the WTK13 and CHB14-2 records, as well as throughout the entire 1900 kyr interval. Comparisons between δDprecip and insolation were performed using June 21st insolation at 20° N, which is based on observations from late Pleistocene and Holocene records demonstrating the sensitivity of eastern African precipitation to this date and latitude 14,60,83,87. We also performed Lomb-Scargle analysis of δD_precip_ to study spectral density of unevenly spaced data with the *plomb* function in MATLAB^98,99^. This method was applied to the two study intervals, 1900–1500 ka and 250–30 ka, which exclude low-resolution intervals. We then used the frequency of the densest spectral peak from each interval (early Pleistocene, 22 kyr; middle to late Pleistocene, 25 kyr; each with bandwidth of ± 5 kyr) to inform gaussian band-pass and notch filtering exercises, which were performed using the time series analysis program AnalySeries version 2.0.8^95^.

## Supplementary Information

Supplementary Information.
