# Supplementary material for: Orbital controls on eastern African hydroclimate in the Pleistocene
Source: Sci Rep. 2022 Feb 24;12:3170. doi: 10.1038/s41598-022-06826-z (PMC8873222; doi:10.1038/s41598-022-06826-z)
Supplement: Supplementary file 1 — Supplementary Information. [file 41598_2022_6826_MOESM1_ESM.docx]

**Supplementary Material**

Orbital controls on eastern African hydroclimate in the Pleistocene

Rachel L. Lupien^1,2^*, James M. Russell^1^, Emma J. Pearson^3^, Isla S. Castañeda^4^, Asfawossen Asrat^5,6^, Verena Foerster^7^, Henry F. Lamb^8,9^, Helen M. Roberts^8^, Frank Schäbitz^7^, Martin H. Trauth^10^, Catherine C. Beck^11^, Craig S. Feibel^12^, Andrew S. Cohen^13^

^1^ Department of Earth, Environmental, and Planetary Sciences, Brown University, Providence, RI 02912

^2^ Biology and Paleo Environment, Lamont-Doherty Earth Observatory of Columbia University, Palisades, NY 10964

^3^ School of Geography, Politics & Sociology, Newcastle University, Newcastle upon Tyne NE1 7RU, UK

^4^ Department of Geosciences, University of Massachusetts Amherst, Amherst, MA 01003

^5^ Department of Mining and Geological Engineering, Botswana International University of Science and Technology, Private Bag 16, Palapye, Botswana

^6^ School of Earth Science, Addis Ababa University, Addis Ababa, Ethiopia

^7^ Institute for Geography Education, University of Cologne, Cologne 50931, Germany

^8^ Department of Geography and Earth Sciences, Aberystwyth University, Aberystwyth SY23 3DB, UK

^9^ Botany Department, Trinity College Dublin, Dublin 2, Ireland

^10^ Institute of Geosciences, University of Potsdam, Potsdam 14476, Germany

^11^Geosciences Department, Hamilton College, Clinton, NY 13323

^12^ Department of Earth and Planetary Sciences, Rutgers University, Piscataway, NJ 08854

^13^ Department of Geosciences, University of Arizona, Tucson, AZ 85721

*Corresponding Author: [rlupien@ldeo.columbia.edu](mailto:rlupien@ldeo.columbia.edu)

Table S1. Isotope data for late Holocene samples from the Turkana and Chew Bahir Basins used for geographic corrections down core.

| *Study Sites* | *Core* | *δD_wax_ (‰)* | *δ^13^C_wax_ (‰)* | *δD_precip_ (‰)* | *reference* |
| --- | --- | --- | --- | --- | --- |
| Turkana | TURK10-14p | -110.5 | -26.1 | -20.2 | Morrissey, 2014^1^ |
| Chew Bahir | CB-05-2010 | -122.3 | -26.7 | -32.0 | Foerster et al., 2014^2^; this study |

**Fig. S1.** WTK13 δD_precip_ before (blue) and after (black with dots) the conservative tuning presented in Lupien et al., 2018. Independent age constraints for WTK13^3,4^ with 1σ analytical error depicted along bottom with symbol indicating dating technique (red star = ^40^Ar/^39^Ar; blue square = magnetostratigraphy).

**Fig. S2.** Leaf wax carbon isotope (δ^13^C_wax_) record from CHB14-2 demonstrates minimal secular trends in the abundance of C_3_ and C_4_ and vegetation but large orbital-scale variability between plant endmembers. With the exception of one ^13^C-enriched outlier, the data indicate mixed C_3_ and C_4_ plant community, with an average of 26% and 74% C_3_ and C_4_, respectively. This is consistent with the modern δ^13^C_wax_ value of -26.7‰, which indicates ~45% C_4_ vegetation. We use this new δ^13^C_wax_ record to correct the new CHB14-2 δD_wax_ record for the impact of plant metabolic pathway (C_3_ or C_4_) on the fractionation of hydrogen and to derive the δD_precip_ record (Fig. S3).

**Fig. S3.** Measured δD_wax_ (left, blue) values from CHB14-2 with δD_precip_ values corrected for plant metabolic pathway (right, orange circles) based on δ^13^C_wax_. The δ^13^C_wax_ data is interpolated to the same depths of the δD_wax_ measurements to produce a δD_precip_ record at the same sampling resolution of the original δD_wax_ measurements (right, orange line). The δD_wax_ and δD_precip_ trends exhibit very little difference, indicating that the metabolic pathway of the plants sourcing leaf waxes to CHB had little effect on the δD_wax_ relative to changes in plant source water δD.

**Fig. S4.** Isotopic corrections of the original δD_wax_ measurements (left axis, orange). Measurements were corrected for vegetation effects with interpolated δ^13^C_wax_ measurements (light blue; Fig. S3) to obtain δD of precipitation (right axis). δD_precip_ was corrected for changes in source water isotopes at different time intervals through an ice volume correction (dark blue). Modern leaf wax isotope anomalizations from each basin were applied downcore to correct for geographic location (teal), and this record is used for all analyses.

**Fig. S5.** June 21^st^ zonal mean 20°N insolation^5^ (top, right) and gaussian 21-kyr ± 5-kyr band-pass filtering of the δD_precip_ study intervals (bottom, black) truncated to 1870-1500 ka and 250–30 ka to omit low sampling resolution sections. As discussed in the main text, June 21^st^ insolation at 20°N is chosen due to its influence on eastern African precipitation in recent geological time. Eccentricity (right axis) highlights the similarity in the modulation of precession-band high- and low-variability packets in both low-latitude insolation and early and middle Pleistocene δD_precip_.

**References**

1. Morrissey, A. Stratigraphic framework and Quaternary paleolimnology of the Lake Turkana Rift, Kenya. *PhD Thesis* vol. PhD (Syracuse University, Syracuse, NY, Paper 62, 2014).

2. Foerster, V. *et al.* 46 000 years of alternating wet and dry phases on decadal to orbital timescales in the cradle of modern humans: the Chew Bahir project, southern Ethiopia. *Climate of the Past* **10**, 977–1023 (2014).

3. Lupien, R. L. *et al.* A leaf wax biomarker record of early Pleistocene hydroclimate from West Turkana, Kenya. *Quaternary Science Reviews* **186**, 225–235 (2018).

4. Sier, M. J. *et al.* The top of the Olduvai subchron in a high-resolution magnetostratigraphy from the West Turkana core WTK13, Hominin Sites and Paleolakes Drilling Project (HSPDP). *Quaternary Geochronology* **42**, 117–129 (2017).

5. Laskar, J. *et al.* A long-term numerical solution for the insolation quantities of the Earth. *Astronomy & Astrophysics* **428**, 261–285 (2004).
